# A safety study of newly generated anti-podoplanin-neutralizing antibody in cynomolgus monkey (*Macaca fascicularis*)

**DOI:** 10.18632/oncotarget.26055

**Published:** 2018-09-07

**Authors:** Takao Ukaji, Ai Takemoto, Ryohei Katayama, Kengo Takeuchi, Naoya Fujita

**Affiliations:** ^1^ Division of Experimental Chemotherapy, The Cancer Chemotherapy Center, Japanese Foundation for Cancer Research, Tokyo, Japan; ^2^ Division of Pathology, The Cancer Institute, Japanese Foundation for Cancer Research, Tokyo, Japan; ^3^ Pathology Project for Molecular Targets, The Cancer Institute, Japanese Foundation for Cancer Research, Tokyo, Japan; ^4^ The Cancer Chemotherapy Center, Japanese Foundation for Cancer Research, Tokyo, Japan

**Keywords:** cynomolgus monkey (Macaca fascicularis), podoplanin, platelet aggregation, tumor metastasis, toxicity

## Abstract

Hematogenous metastases are enhanced by platelet aggregation induced by tumor cell-platelet interaction. Podoplanin is a key molecule to enhance the platelet aggregation and interacts with C-type lectin-like receptor 2 (CLEC-2) on platelet *via* PLAG domains. Our previous reports have shown that blocking podoplanin binding to platelets by neutralizing antibody specific to PLAG4 domain strongly reduces hematogenous metastasis. However, podoplanin is expressed in a variety of normal tissues such as lymphatic vessels and the question remains whether treatment of tumors with anti-podoplanin neutralizing antibodies would be toxic. Monkeys are the most suitable species for that purpose. PLAG3 and PLAG4 domains had high homology among various monkey species and human. PLAG domain deleted mutants were indicated that monkey PLAG4 domain played a more crucial role in podoplanin-induced platelet aggregation than did the PLAG3 domain as in human. Moreover, newly established neutralizing antibodies (1F6, 2F7, and 3F4) targeting the monkey PLAG4 domain blocked interaction between monkey podoplanin and CLEC-2. Especially, the 2F7 neutralizing antibody strongly suppressed platelet aggregation and pulmonary metastasis. Furthermore, inhibiting podoplanin function with 2F7 neutralizing antibody exhibited no acute toxicity in cynomolgus monkeys. Our results suggested that targeting podoplanin with specific neutralizing antibodies may be an effective anticancer treatment.

## INTRODUCTION

Although hematogenous metastasis of cancer cells is occurred by the migration of detached tumor cells from the primary site into the blood vessel, most cancer cells that infiltrate blood vessels are rapidly eliminated. Less than 0.1% of cancer cells survive for 24 h in the bloodstream [1]. Platelets are a critical component that affects tumor cell survival within the vascular system and enhances the metastasis formation [2]. Platelets facilitate the survival of circulating tumor cells by forming large tumor cell–platelet aggregates. These aggregates protect the tumor cells from immunological assault or shear stress, upregulate tumor malignancy by releasing soluble factors from activated platelets, enhance embolization in the microvasculature, and result in metastatic foci in distant organs. The importance of platelets in tumor metastasis is also supported by the fact that anti-platelet agents and thrombocytopenia reduce the incidence of metastasis in some experimental models [3, 4]. The administration of anti-coagulants reportedly lowers the mortality rate [5, 6]. These findings strongly suggest that activation of platelets is intricately involved in hematogenous metastasis. Blocking platelet-tumor interaction might therefore make a significant contribution to reducing cancer mortality.

Podoplanin, also known as Aggrus, T1 alpha [7] and gp36 [8] is a type I transmembrane sialoglycoprotein and a key molecule in pathologic platelet aggregation and activation [9]. Podoplanin induces platelet aggregation by binding directly to C-type lectin-like receptor 2 (CLEC-2) [10, 11]. Podoplanin binding to CLEC-2 transmits platelet activation signals to the Src family kinases, Syk and phospholipase Cγ2 [12, 13]. Podoplanin contains a characteristic conserved EDXXVTPG motif (where X may be any amino acid) in its extracellular domain. This motif is critical for podoplanin-mediated platelet aggregation. In a previous report, we designated this motif as the platelet aggregation-stimulating (PLAG) domain [9]. In that study, we found that podoplanin possesses three tandem repeats of the PLAG domain (PLAG1–3). However, we recently identified an additional PLAG4 domain which includes the highly conserved EDXXT motif and is closely related to the other PLAG domains [14]. Crystal structural analysis of the PLAG3 domain complexed with CLEC-2 revealed that the acidic side chains of Glu^47^ and Asp^48^ and the sialic acid attached to Thr^52^ on PLAG3 were recognized by basic residues on CLEC-2 [15]. We then further reported that Glu^81^, Asp^82^, and Thr^85^ residues in PLAG4 were indispensable for binding to CLEC-2 [14].

Many reports have indicated that anti-podoplanin neutralizing antibodies that block PLAG domain binding to CLEC-2 can suppress hematogenous metastasis. We generated a neutralizing antibody, MS-1, which specifically recognizes the PLAG3 domain in human podoplanin. It suppressed podoplanin-induced platelet aggregation and hematogenous metastasis in a mouse model [11]. More recently, we generated PG4D2, a neutralizing antibody recognizing the PLAG4 domain in human podoplanin and found that it strongly suppressed podoplanin–CLEC-2 binding and podoplanin-mediated hematogenous metastasis of human podoplanin-expressing tumor cells inoculated into mice [14]. On the other hand, a different reported approach has been to block podoplanin–CLEC-2 interaction by targeting the CLEC-2 receptor itself. CLEC-2 depletion by means of an anti-CLEC-2 antibody suppressed experimental hematogenous tumor metastasis and tumor growth, inhibited thrombus formation in the lungs, and improved the outcome in tumor-bearing mice [16]. Using a mouse xenograft model, Chang et al. [17] synthesized 2CP, a small compound that specifically binds to CLEC-2 and inhibits podoplanin-induced platelet aggregation in tumor cells without affecting normal hemostasis. Moreover, soluble factors such as PDGF or TGF-β secreted from podoplanin-activated platelets have been shown to induce the growth of some tumors [18, 19]. The blocking podoplanin–CLEC-2 interaction by neutralizing antibody contributes to suppress the tumor cell growth [11]. Based on this accumulating evidence, podoplanin appears to be a promising therapeutic target for metastatic tumors. Moreover, CLEC-2–deficient platelets have been reported to respond normally to platelet agonists, such as collagen, ADP, U46619, and PAR-4, suggesting that inhibition of podoplanin–CLEC-2 interaction may not adversely affect physiologic hemostasis [20]. Such properties are vital if this type of antibody is to be used safely as part of an anticancer therapeutic regimen.

Podoplanin is expressed in a variety of normal tissues such as lymphatic vessels, kidney podocytes, mesothelium, and alveolar epithelium. In the absence of podoplanin, the perinatal development of lung and lymphatics is lethally hindered [21, 22]. However, it has been demonstrated in podoplanin-conditional knockout mice that there are no serious effects of podoplanin deletion after birth [23]. The question remains whether treatment of tumors with anti-podoplanin neutralizing antibodies would be toxic. Therefore, possible adverse effects and toxicities must be investigated before such antibodies can be tested clinically as antitumor agents.

The cynomolgus monkey (*Macaca fascicularis*) is frequently used for biopharmaceutical toxicity studies because it commonly exhibits pharmacologic responses resembling those in humans [24]. The result of nonclinical studies using monkeys is an important step before clinical testing. No toxicity studies have yet been undertaken in monkeys testing anti-podoplanin neutralizing antibodies.

This study was designed to investigate the functional PLAG domain in monkey podoplanin and the safety of anti-podoplanin neutralizing antibody administration. Experiments were performed using PLAG domain-deleted monkey mutants and with the anti-podoplanin neutralizing antibody that recognizes the monkey podoplanin PLAG4 domain.

## RESULTS

### Involvement of the monkey podoplanin PLAG4 domain in platelet aggregation

First, we selected podoplanin sequences of various species including closely related monkeys to human such as chimpanzee and gorilla [25] from the NCBI GenBank and compared them. A phylogenetic tree based on podoplanin protein sequences revealed that monkey podoplanin formed a monophyletic cluster with that of humans and was well-supported by high bootstrap values (Supplementary Figure 1A). The cynomolgus monkey podoplanin sequence was 92.6% identical to that of human podoplanin at the amino acid level. Moreover, protein sequences in the PLAG3 (47-EDDVVTPG-54) and PLAG4 (81-EDLPT-87) domains had high homology among various monkey species and completely matched human podoplanin. Glu^47^, Asp^48^, and Thr^52^ residues on PLAG3 and Glu^81^, Asp^82^, and Thr^85^ residues on PLAG4, critical residues for human podoplanin-CLEC-2 binding, were conserved among these species (Supplementary Figure 1B). To evaluate the contributions of both domains to CLEC-2 binding, we substituted commercially available recombinant human or mouse CLEC-2 for monkey CLEC-2. The full amino acid sequence of monkey CLEC-2 was 93.4 % and 64.8 % identical to the human and mouse sequences, respectively. On the other hand, another group reported that four arginine residues (Arg^107^, Arg^118^, Arg^152^ and Arg^157^) on human CLEC-2 were critical for human podoplanin binding to CLEC-2 at two positions: the interaction of the PLAG3 Glu^47^-Asp^48^ doublet with CLEC-2 Arg^107^, Arg^152^, and Arg^157^ and of PLAG3 sialic acid attached to Thr^52^ with CLEC-2 Arg^118^ [15]. In monkey CLEC-2, Lys^118^ was substituted for Arg^108^, while the Arg^107^, Arg^152^ and Arg^157^ residues were conserved (Supplementary Figure 1C).

**Figure 1:**
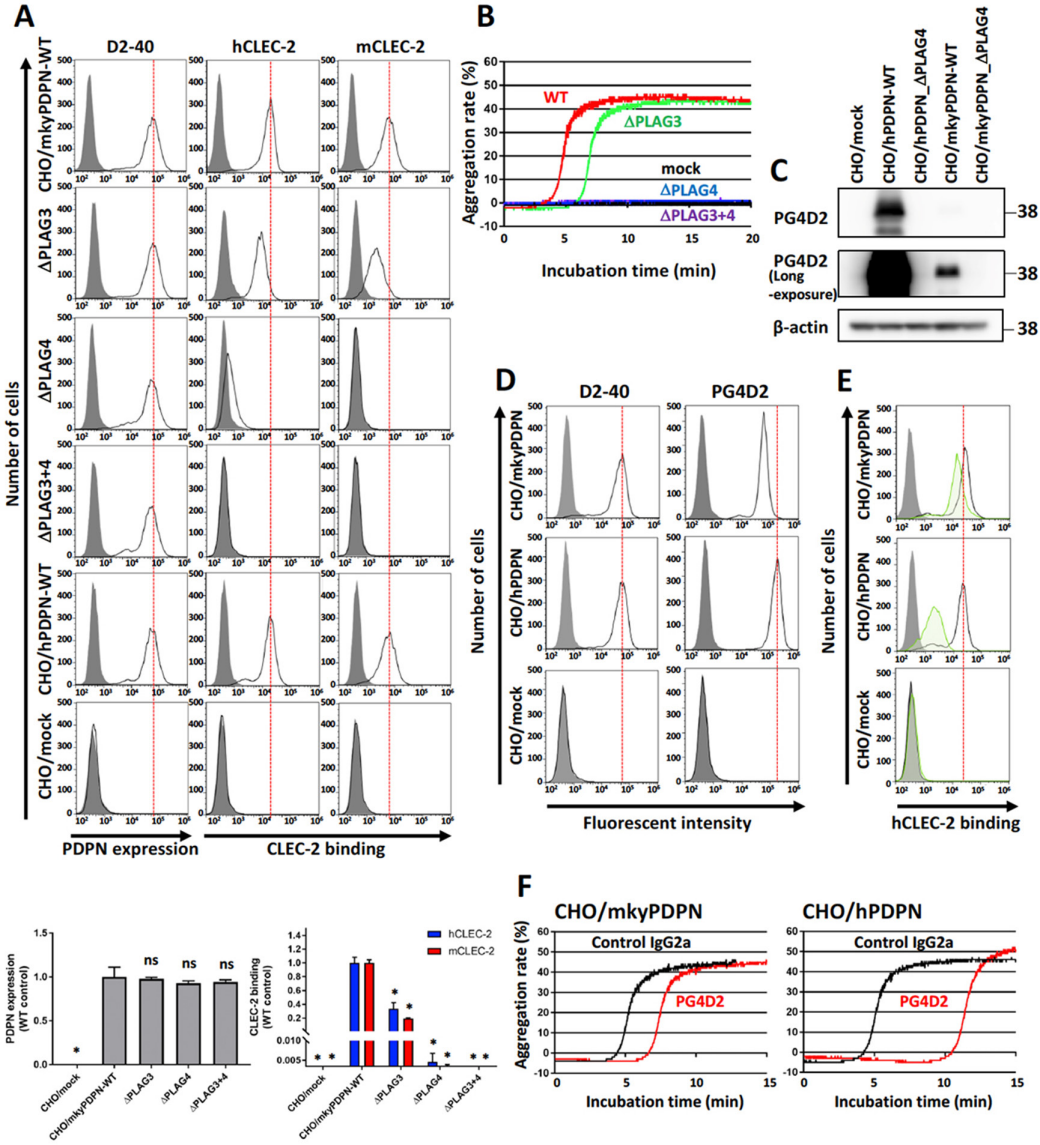
PLAG4 domain contributes to monkey podoplanin-induced platelet aggregation **(A)** CHO cells stably transfected with an empty vector (mock), wild-type monkey podoplanin (mkyPDPN-WT), wild-type human podoplanin (hPDPN-WT), or PLAG domain-deleted podoplanin mutants (mkyPDPN−ΔPLAG3, −ΔPLAG4, and −ΔPLAG3+4) were treated with PBS (closed areas) or anti-podoplanin mAb (D2-40; open areas) to measure PDPN expression levels. The ability to bind with human or mouse C-type lectin-like receptor 2 (CLEC-2) was compared with PBS (closed areas), hCLEC-2-(His)_10_ (0.4 μg/ml), or mCLEC-2-(His)_10_ (5 μg/ml) (open areas). After washing, cells were incubated with Alexa Flour 488-conjugated second antibody. Flow cytometry data (upper) and quantitative graphs (lower) are shown. Each value in the lower graphs is the mean ± SD (N = 3) of peak values normalized to that of CHO/PDPN-WT. ^*^P < 0.05 using Mann–Whitney *U* test. ns, not significant. **(B)** Transfected CHO cells were incubated with mouse platelet-rich plasma (PRP). The platelet aggregation rate was estimated using an aggregometer. **(C)** CHO transfected with empty vector (mock), hPDPN-WT, hPDPN−ΔPLAG4, mkyPDPN, or mkyPDPN−ΔPLAG4 cells were lysed and immunoblotted with PG4D2 antibodies to PDPN or with β-actin. **(D)** Transfected CHO cells were treated with PBS (closed areas) or anti-PDPN antibodies D2-40 or PG4D2 (1 μg/ml) (open areas), and fluorescence intensity was measured. After washing, cells were incubated with Alexa Flour 488-conjugated second antibody. **(E)** Transfected CHO cells were incubated with 100 μg/mL of control IgG2a or anti-podoplanin antibody PG4D2, followed by incubation with 0.4 μg/mL of hCLEC-2-(His)_10_ protein (open areas: control IgG2a-treated samples; green areas: PG4D2-treated samples). After washing, cells were further incubated with Alexa Flour 488-conjugated anti-penta-His second antibody. CLEC-2 binding was measured by flow cytometry. Gray areas indicate the fluorescence intensity of samples not treated with CLEC-2. **(F)** CHO/mkyPDPN-WT or CHO/hPDPN-WT cells were incubated with 10 μg/mL of control IgG2a or PG4D2 followed by incubation with mouse PRP. The aggregation rate was estimated using an aggregometer.

We assessed the ability of the transfected CHO cells (CHO/mkyPDPN-WT, CHO/mkyPDPN-ΔPLAG3, -ΔPLAG4, or -ΔPLAG3+4) to bind to recombinant human or mouse CLEC-2. The total podoplanin expression level in CHO/mkyPDPN-WT, its deleted mutants, or CHO/hPDPN-WT was estimated using a human anti-podoplanin mAb D2-40, a potential diagnostic marker for many different types of human tumors that recognizes the PLAG1 and PLAG2 domains (33–44 aa) [26]. We selected clones possessing similar podoplanin expression signals by flow cytometric analysis (Figure 1A, left panels). The binding ability of mkyPDPN-WT to human and mouse CLEC-2 was almost same as that of hPDPN-WT (Figure 1A middle and right panels). The ΔPLAG3 mutant significantly attenuated PDPN binding to hCLEC-2 and mCLEC-2, but only showed a partial reduction of its binding capability. On the other hand, the ΔPLAG4 mutant almost completely suppressed PDPN binding to hCLEC-2 and completely abrogated to mCLEC-2, significantly. The ΔPLAG3+4 mutant completely abrogated the binding capability to both CLEC-2 (Figure 1A, middle and right panels). Consistent with CLEC-2-binding activity, the ΔPLAG3 mutant had lower platelet-aggregating ability compared with WT. Both the ΔPLAG4 and ΔPLAG3+4 mutant had no platelet-aggregating ability (Figure 1B). These results indicate that the monkey PLAG4 domain played a more crucial role in podoplanin-induced platelet aggregation than did the PLAG3 domain.

Next, we estimated the cross-reactivity of PG4D2 with monkey podoplanin to evaluate whether acute toxicity testing with PG4D2 was feasible in monkeys. PG4D2, the neutralizing antibody specific for the human PLAG4 domain, recognized the perimeter structure from Arg^79^ to Leu^83^ (79-RIEDL-83) in human podoplanin. The homologous region in cynomolgus monkey podoplanin had His^79^ residue substituted for Arg^79^ (Supplementary Figure 1B). PG4D2 reactivity against CHO/mkyPDPN was much lower than against CHO/hPDPN (Figure 1C, 1D right panels). Masking the PLAG4 domain with PG4D2 attenuated monkey podoplanin binding to human CLEC-2, but this suppressive effect was much lower than that in CHO/hPDPN (Figure 1E). PG4D2 treatment delayed the platelet aggregation induced by the interaction of CHO/mkyPDPN with mouse platelets but with a lower suppressive effect than of human podoplanin (Figure 1F). By contrast, the MS-1 mAb reacted with both monkey and human podoplanin PLAG3 domains and suppressed monkey podoplanin-induced platelet aggregation (Supplementary Figure 2B and 2C).

### Establishment of antibodies recognizing the PLAG4 domain in monkey podoplanin

**Figure 2:**
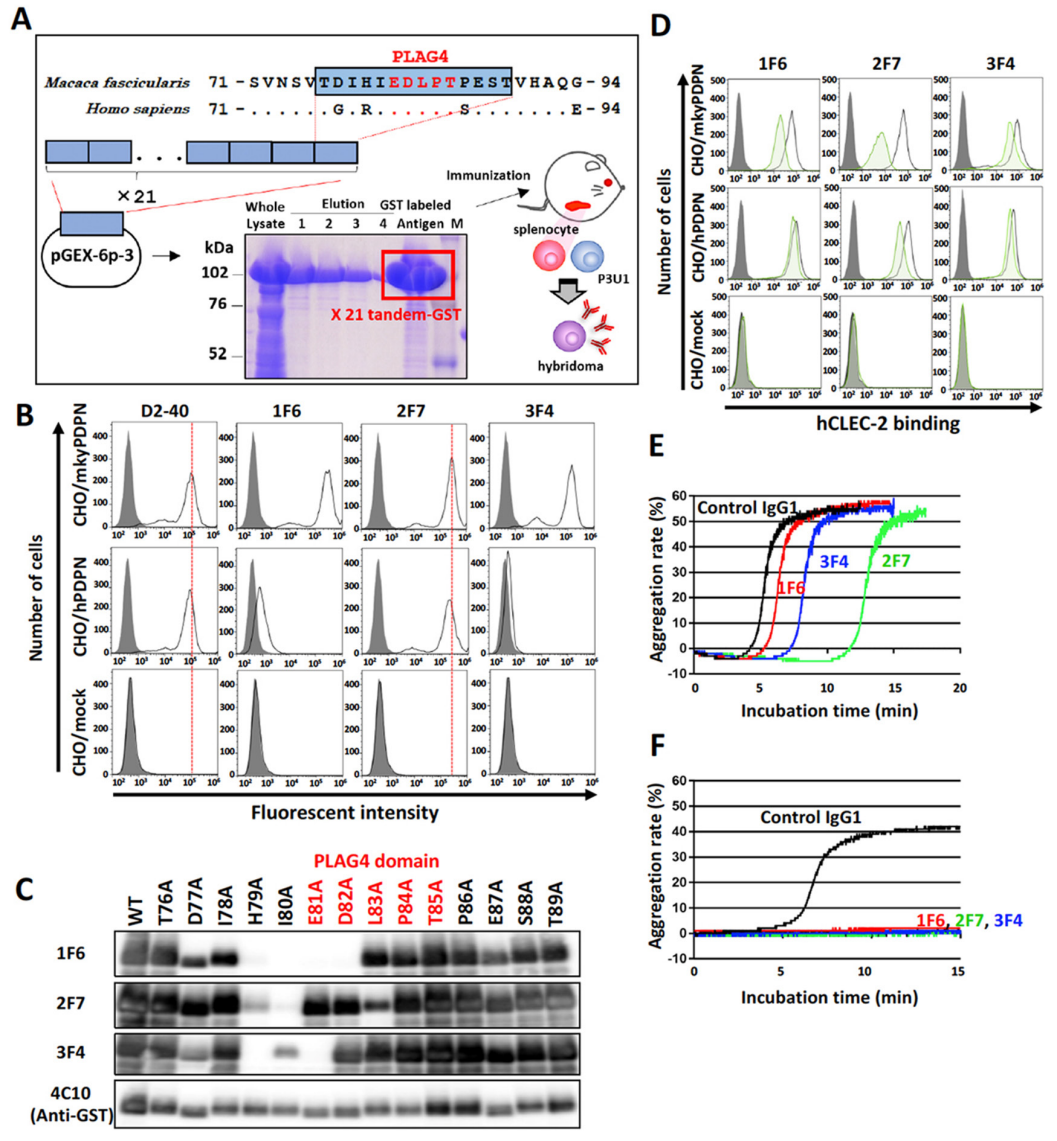
Establishment of monkey podoplanin-neutralizing antibodies **(A)** Purification of a monkey podoplanin (mkyPDPN) immunogen to establish a hybridoma secreting anti-PDPN monoclonal antibodies (mAbs). A mkyPDPN cDNA region encoding amino acids 76–89 (226–267 bp) was tandemly connected 21 times. The cDNA fragment was inserted into a pGEX-6P-3 vector, and a glutathione S-transferase (GST)-tagged mkyPDPN peptide (76–89 aa) produced by BL21 (DE3) *E. coli* was purified using glutathione sepharose. BALB/c mice were injected with the GST-tagged peptide, after which their splenocytes were fused with mouse myeloma P3U1 cells using polyethylene glycol. Hybridoma screening and antibody purification from ascites were performed. **(B)** CHO cells transfected with an empty vector (mock), wild-type monkey podoplanin (mkyPDPN-WT), wild-type human podoplanin (hPDPN-WT) were treated with PBS (closed areas) or antibodies (open areas), including anti-PDPN mAb D2-40, 1F6, 2F7, or 3F4 to measure PDPN expression levels. **(C)** GST-tagged recombinant mkyPDPN protein (WT) and its point mutants were expressed in *E. coli*. Cell lysates were electrophoresed and immunoblotted with antibodies to PDPN (1F6, 2F7, 3F4) or GST. The PLAG4 domain is indicated by red letters. **(D)** CHO/mock, CHO/mkyPDPN-WT, or CHO/hPDPN-WT cells were incubated with 100 μg/mL of control IgG1 or anti-PDPN antibodies 1F6, 2F7, or 3F4, followed by incubation with 1 μg/mL of hCLEC-2-(His)_10_ (open areas: control IgG-treated samples; green areas: anti-PDPN mAb-treated samples). After washing, cells were further incubated with Alexa Flour 488-conjugated anti-penta-His second antibody. CLEC-2 binding was measured by flow cytometry. Gray areas indicate the fluorescence intensity of samples not treated with CLEC-2. **(E)** CHO/mkyPDPN-WT cells were incubated with 10 μg/mL of control IgG1, 1F6, 2F7, or 3F4 mAbs followed by incubation with mouse platelet-rich plasma (PRP). The aggregation rate was estimated using an aggregometer. **(F)** PLAG3 domain-deleted mkyPDPN mutant cells were incubated with 10 μg/mL of control IgG1, 1F6, 2F7, or 3F4 mAbs, followed by incubation with mouse PRP. The aggregation rate was estimated using an aggregometer.

To further investigate the role of the PLAG4 domain in podoplanin-induced platelet aggregation in monkeys, we established mAbs to recognize the PLAG4 domain and neutralize the interaction between monkey podoplanin and CLEC-2. A tandemly repeated cynomolgus monkey PLAG4 peptide (76-TDIHIEDLPTPEST-89) was purified as an antigen from a bacteria lysate and injected into mice. After several times of immunization, the splenocytes were collected from mice and fused with P3U1 myeloma cells (Figure 2A). Finally, three hybridoma cell lines secreting anti-PLAG4 mAbs were established. The three mAbs (1F6, 2F7, and 3F4) strongly recognized intact monkey podoplanin, and 2F7 mAb in particular strongly recognized human podoplanin (Figure 2B).

Epitope mapping of these mAbs was established by their reactivity to recombinant monkey podoplanin lysate expressed in *E. coli* that harbored point mutations of each amino acid to alanine. We confirmed that these mAbs recognized the perimeter structure from Asp^77^ to Asp^82^ (1F6), from His^79^ to Leu^83^ (2F7), and from Asp^77^ to Glu^81^ (3F4). All these mAbs thus partly masked the PLAG4 domain (Figure 2C) and interfered with the interaction between monkey podoplanin and human CLEC-2. The 2F7 mAbs strongly suppressed monkey podoplanin binding to human CLEC-2 and partially suppressed the interaction of human podoplanin with human CLEC-2 (Figure 2D). Moreover, these mAbs strongly suppressed the interaction between monkey podoplanin and mouse CLEC-2, and 2F7 almost completely suppressed human podoplanin binding to mouse CLEC-2 (Supplementary Figure 3). The platelet aggregation induced by CHO/mkyPDPN cells was strongly suppressed by 2F7 and slightly so by 3F4, with minimal effect by 1F6 (Figure 2E). Additionally, the platelet aggregation induced by mutated CHO/mkyPDPN−ΔPLAG3 was completely suppressed by all three mAbs (Figure 2F).

### Anti-PLAG4 antibodies suppressed endogenous podoplanin-induced platelet aggregation and pulmonary metastasis

**Figure 3:**
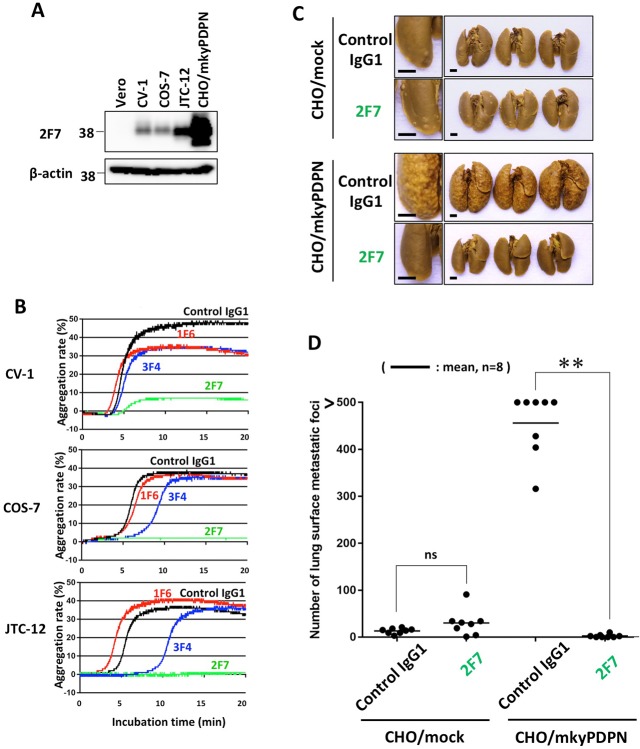
Inhibition of endogenous podoplanin-induced platelet aggregation and podoplanin-mediated pulmonary metastasis by anti-PLAG4 mAb **(A)** CHO cells transfected with wild-type monkey podoplanin (CHO/mkyPDPN-WT) and Vero, CV-1, COS-7 or JTC-12 cells were lysed and immunoblotted with antibodies to PDPN (1F6, 2F7 or 3F4) or with β-actin. **(B)** CV-1, COS-7, or JTC-12 cells were incubated with 10 μg/mL of control IgG1, 1F6, 2F7 or 3F4, followed by incubation with mouse platelet-rich plasma to assess platelet aggregation. **(C)** BALB/c-*nu*/*nu* mice were intravenously injected with 10 μg/mouse of control IgG1 or 2F7. The next day, CHO/mock or CHO/mkyPDPN-WT (2.5 × 10^5^ cells/mouse) were intravenously inoculated into the mice. The mice were euthanized 16 days later, and lung surface metastatic foci were counted. Representative pictures are shown. Bars, 2 mm. **(D)** Numbers of metastatic. Bars, mean (N = 8). ^**^P < 0.01 by a Mann–Whitney *U* test. ns, not significant.

To evaluate the role of the PLAG4 domain in endogenous podoplanin-expressing cells, we used three monkey kidney cell lines, CV-1, COS-7 and JTC-12 (Figure 3A). The mAbs 2F7 strongly suppressed platelet aggregation induced by all three lines, but 1F6 did not. (Figure 3B).

To verify the role of the PLAG4 domain in monkey, we performed an *in vivo* metastasis assay using the 2F7 mAb, which had most strongly suppressed podoplanin binding to CLEC-2. Podoplanin-induced pulmonary metastasis was significantly blocked by prior administration of 2F7 mAb (Figure 3C and 3D).

### Acute toxicity testing of anti-podoplanin neutralizing antibody in *Macaca fascicularis*

**Figure 4:**
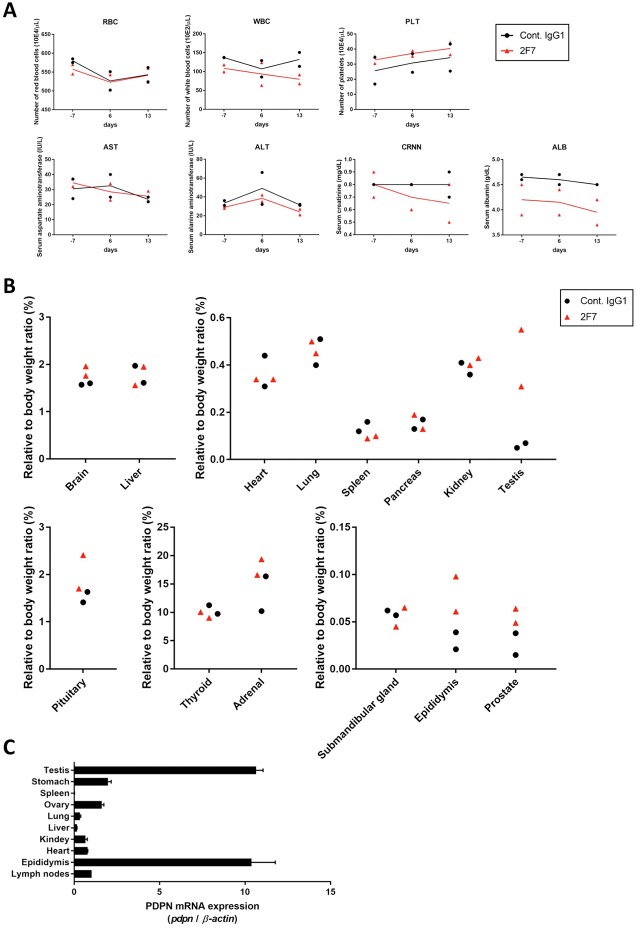
Evaluation of toxicity of anti-podoplanin neutralizing antibodies in *Macaca fascicularis* **(A)** Monkeys were intravenously injected with 100 mg/kg of control IgG1 or 2F7 antibodies. Blood tests were performed 7 days before and 6 and 13 days after injection. Normal range for each parameter was shown as mean (-2SD/ +2SD). RBC (red blood cells), 576 (497/ 654) 10E4/μL; WBC (white blood cells), 116.7 (40.6/ 192.8) 10E2/μL; PLT (platelets), 35.0 (19.6/ 50.5) 10E4/μL; AST (aspartate aminotransferase), 35 (14/ 56) IU/L; ALT (alanine aminotransferase), 40 (7/ 74) IU/L; CRNN (creatinine), 0.69 (0.39/ 0.99) mg/dL; ALB (albumin), 4.3 (3.8/ 4.8) g/dL. **(B)** Two weeks after injection, monkeys were euthanized and tissue and organ weights were measured. **(C)** Podoplanin (PDPN) mRNA in normal tissues of *Macaca fascicularis* estimated by quantitative real time PCR. The expression level of each PDPN mRNA was normalized to that of *β-actin*.

**Figure 5:**
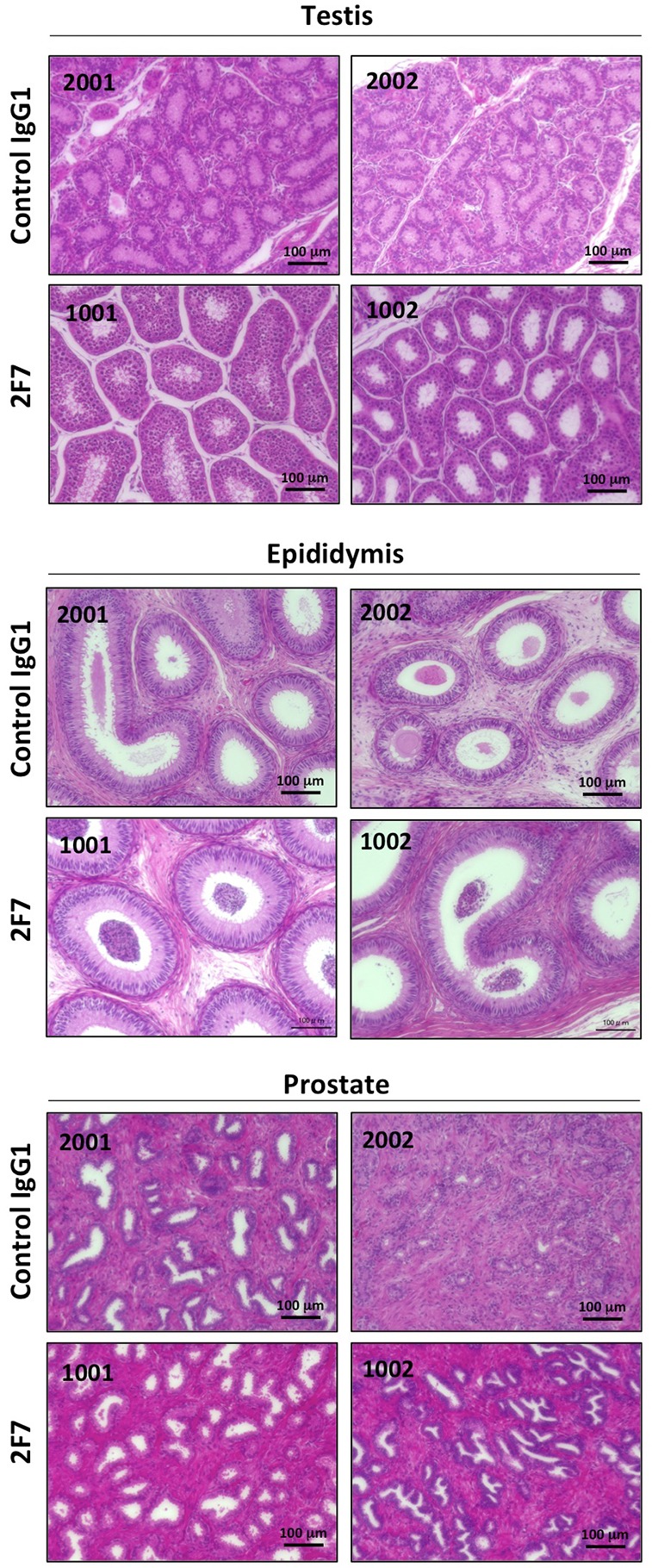
Histopathological examination after injection of anti-podoplanin neutralizing antibodies in *Macaca fascicularis* Representative images of sections of the testis, epididymis, and prostate tissue from monkeys injected with control IgG1 (identity number: 2001 and 2002) or anti-podoplanin antibody 2F7 (identity number: 1001 and 1002). Each section was treated with hematoxylin-eosin.

**Figure 6:**
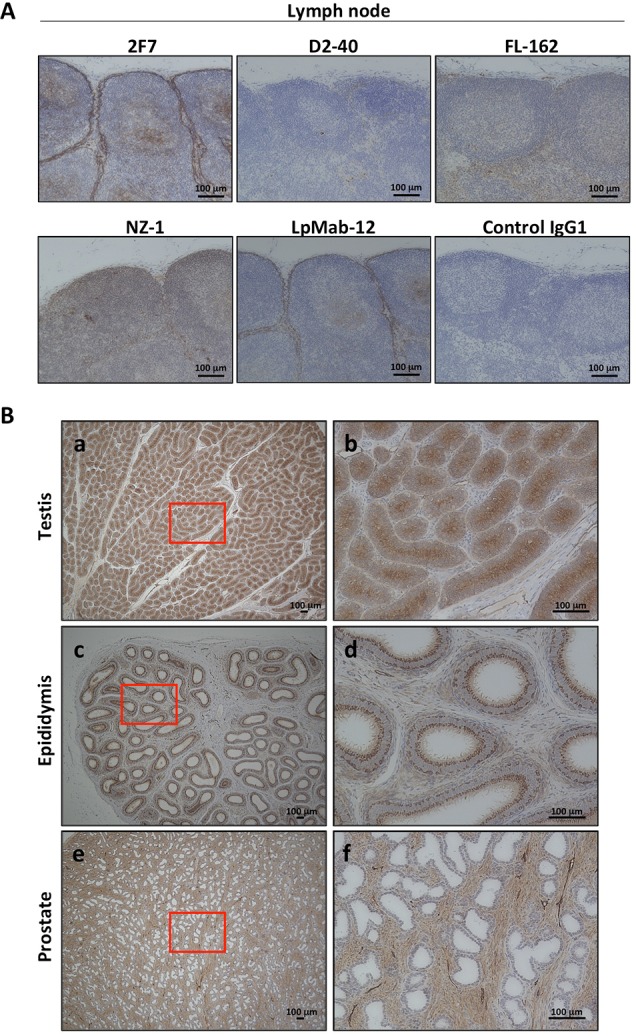
Podoplanin expression in normal tissue of *Macaca fascicularis* by immunohistochemical staining **(A)** Detection methods of podoplanin expression in lymph node were evaluated by anti-podoplanin antibodies: 2F7, D2-40, FL-162, NZ-1, LpMab-12, or control IgG1, respectively. Only 2F7 and LpMab-12 were recognized the podoplanin-expressing cells. Representative images of immunohistochemical staining were shown. **(B)** Testis **(a** and **b)**, epididymis **(c** and **d)**, or prostate **(e** and **f)** in *Macaca fascicularis* were immunolabeled with 2F7 for localization of podoplanin and representative images were shown. The higher magnification of area surrounded by red line (a, c or e) was shown in b, d, or f, respectively.

To evaluate the toxicity of anti-podoplanin antibody blocking of podoplanin-CLEC-2 binding, we prepared a large amount of purified 2F7 antibody and control IgG1 (Supplementary Figure 4A). Cynomolgus monkeys were injected with these antibodies and then evaluated for toxic effects (Supplementary Figure 4B). Hematologic and biochemical analysis of blood samples revealed no difference between those receiving the podoplanin-neutralizing antibody and the control IgG1 monkeys (Figure 4A, and Supplementary Figure 5A and 5B). The relative weights of the brain, pituitary, thyroid, submandibular gland, heart, lung, liver, spleen, pancreas, kidney, and adrenal did not differ significantly between the two groups. However, the weights of the testis, epididymis, and prostate in the 2F7 group were higher than those of the control IgG1 group (Figure 4B). Additionally, podoplanin was strongly expressed in the monkeys’ reproductive organs including the ovary, testis and epididymis (Figure 4C). Histologic evaluation with hematoxylin and eosin staining of tissue specimens from the two groups did not differ (Figure 5, only testis, epididymis and prostate are shown). Monkeys in the podoplanin-neutralizing antibody group were mature, but the monkeys in the control IgG1 group seemed to be immature. 2F7 strongly recognized surface of cortical nodule and follicular dendritic cells of germinal center in lymph node as well as LpMab-12. On the other hand, monkey podoplanin was not immunolabeled by D2-40, FL-162 and NZ-1 (Figure 6A). Podoplanin expression was detected by 2F7 in convoluted seminiferous tubules of testis (Figure 6Ba and 6Bb), ducts of epididymis (Figure 6Bc and 6Bd), and interstitial connective tissue of prostate (Figure 6Be and 6Bf), but not by control IgG1 (Supplementary Figure 6).

## DISCUSSION

Podoplanin overexpression has been detected in wide range of tumor cells, including squamous cell carcinoma [27], mesothelioma [28], glioblastoma [29], bladder cancer [30], angiosarcoma [31], brain tumors [32], testicular seminoma [33], and osteosarcoma [34]. It is well recognized that podoplanin expression is correlated with a poor prognosis and tumor malignancy in lung carcinomas, oral squamous cell carcinomas, and breast cancers [35–39]. Interference with podoplanin-induced platelet aggregation and activation is directly associated with inhibition of podoplanin-mediated tumor metastasis [40]. As described above, a neutralizing antibody targeting the podoplanin PLAG domain is a strong candidate to target podoplanin-induced hematogenous metastasis. Because our previously established neutralizing antibodies suppress interaction between podoplanin and CLEC-2, they are capable of inhibiting hematogenous metastasis of podoplanin-expressing cancer cells [11, 14]. Although there have been many reports of podoplanin-neutralizing antibodies capable of inhibiting podoplanin–CLEC-2 binding, the toxicity associated with such antibodies has not been investigated.

To identify PLAG domains critical for CLEC-2 binding in monkeys. We used human and mouse CLEC-2 to evaluate the interaction between monkey podoplanin and CLEC-2. Both human and monkey CLEC-2 cross-reacted with mkyPDPN-WT at the same signal intensity as with hPDPN-WT (Figure 1A middle and right panel). The reason for the cross-reactivity with monkey podoplanin is likely the fact that the critical residues (Glu^47^, Asp^48^, and Thr^52^ residues on PLAG3 and Glu^81^, Asp^82^, and Thr^85^ residues on PLAG4) on human podoplanin that bind human CLEC-2 were completely conserved on monkey podoplanin (Supplementary Figure 1B). Moreover, 3 of 4 Arg residues on CLEC-2 critical for podoplanin binding in humans were also conserved on monkey CLEC-2 (Supplementary Figure 1C). Mouse CLEC-2 also cross-reacted with monkey podoplanin. In previous research, we substituted mouse platelets for human podoplanin-induced platelet aggregation assays because mouse CLEC-2 cross reacts with human podoplanin. Because monkey podoplanin amino acid sequences were 92.6% homologous with human podoplanin, we speculated that monkey podoplanin might also bind mouse CLEC-2. We therefore demonstrated that either human or mouse CLEC-2 could be substituted for monkey CLEC-2 to evaluate the interaction between monkey podoplanin and CLEC-2.

Deletion of the PLAG3 or PLAG4 domains significantly attenuated CLEC-2 binding and monkey podoplanin-induced platelet aggregation, an effect especially obvious with DPLAG4 mutants, with almost complete loss of CLEC-2 binding and platelet aggregation (Figure 1A and 1B). Therefore, we demonstrated that the PLAG3 and PLAG4 domains in the monkey, and particularly PLAG4, play a crucial role in podoplanin-induced platelet aggregation as they do in humans. The PG4D2 neutralizing antibody specific to the human PLAG4 domain was not suitable for assessing the toxicity of podoplanin inhibition in monkeys because it had less affinity for monkey podoplanin and barely suppressed monkey podoplanin-induced platelet aggregation (Figure 1D and 1F). On the other hand, our newly established neutralizing antibodies (1F6, 2F7, and 3F4) targeting the monkey PLAG4 domain blocked interaction between monkey podoplanin and CLEC-2. The 2F7 neutralizing antibody could inhibit platelet aggregation induced by CHO cells expressing monkey podoplanin and by monkey podoplanin endogenously expressing cell lines (Figure 2E and 3B). It also strongly suppressed the pulmonary metastasis of CHO cells expressing monkey podoplanin (Figure 3C and 3D). In our previous results obtained by using several anti-podoplanin neutralizing antibodies, pulmonary metastasis of cancer cells was suppressed by the intravenous injection of anti-podoplanin neutralizing antibody on 1 hour to 3 days before tumor injection. On the other hand, the suppressive effects of antibodies became less remarkable when the administration was on 1 to 7 days after injection. We suggest that administration on 1 to 3 days before cell injection will maximize their metastasis-inhibitory effects. Each neutralizing antibody partially masked the PLAG4 domain, but 2F7 most strongly suppressed its binding to CLEC-2. 2F7 recognizes the same region as the PG4D2 epitope, i.e., from His^79^ to Leu^83^ (79-HIEDL-83) of monkey podoplanin in 2F7 and Arg^79^ to Leu^83^ (79-RIEDL-83) of human podoplanin in PG4D2 (Figure 2C). Therefore, masking the perimeter structure on podoplanin from 79 to 83 aa, which included the PLAG4 domain, appears to induce strong suppression of binding. These findings indicate that interaction between monkey podoplanin and CLEC-2 can be suppressed by PLAG4 neutralizing antibodies. The PLAG4 domain in the monkey has as crucial a role in podoplanin-mediated hematogenous metastasis as in humans.

Having demonstrated the efficacy of the antibodies we developed to neutralize podoplanin function, we conducted acute toxicity testing in cynomolgus monkeys given excess amounts of the podoplanin-neutralizing antibodies. As noted above, podoplanin expression is reportedly expressed on lymphatic endothelial cells [31], kidney podocytes [41, 42], mesothelium, and alveolar epithelium. Podoplanin helps to separate the blood and lymphatic vascular systems and regulates the development of lymphatic vessels [13, 22, 43]. Podoplanin acts to regulate the initial stage of osteocyte differentiation [23]. It serves as a marker of nonpathogenic Th17 cells that have lost IL-17 secretion and functions as a negative regulator of Th17-induced inflammation in humans [44]. The toxicity testing did not reveal significant differences in hematology and biochemistry testing between the control group and the group receiving the 2F7 anti-podoplanin neutralizing antibody. Moreover, although we expected to generate mouse IgG-specific immune response in monkey, no influence caused by administration of control mouse IgG1 was exhibited in hematological and blood analysis or histopathology examination. Therefore, it was suggested that the immune response against mouse IgG was not strongly induced as the toxicity was reflected. On the other hand, reproductive tissues from the monkeys treated with antibody, including testis, epididymis, and prostate, weighed more than those in control monkeys. Higher podoplanin expression was also detected in these reproductive tissues in monkeys (Figure 4C). We previously reported that podoplanin mRNA is highly expressed in the human testis and prostate [30]. Moreover, many podoplanin-expressing cells could be detected by 2F7 in testis, epididymis and prostate (Figure 6B). Therefore, the relatively heavier reproductive tissues might have been caused by the podoplanin-neutralizing antibody. However, on histologic analysis, the difference in tissue weight was found to be due to greater sexual maturity in the antibody-treated monkeys rather than the inhibition of podoplanin (Figure 5). Cynomolgus monkeys vary considerably in the age at which sexual maturity is achieved, so either mature or immature testes can be observed in animals three to five years of age. Neither body weight nor age are predictive of the degree of testicular maturity that is seen histologically [45]. The accessory reproductive organs, i.e., the epididymis and prostate, develop once the testis reach sexual maturity [46]. Although we randomly assigned monkeys to either the control group or the antibody-treated group, we believe it was a coincidence that the 2F7 treated group was more sexually mature than the control group. Regardless, our findings indicate that inhibition of podoplanin by the neutralizing antibody did not result in any acute toxicity in the monkeys.

In this study, we demonstrated that monkey podoplanin can induce platelet aggregation by binding CLEC-2, with the PLAG4 domain prominently involved as it is in humans. Inhibiting podoplanin with a neutralizing antibody targeting the PLAG4 domain exhibited no acute toxicity in cynomolgus monkeys. Our findings strongly suggest that podoplanin is a promising therapeutic target for treating cancer. A neutralizing antibody blocking podoplanin may be particularly useful in suppressing hematogenous metastasis.

## MATERIALS AND METHODS

### Multiple sequence alignment

Podoplanin and CLEC-2 sequences from various species were obtained from NCBI (https://www.ncbi.nlm.nih.gov/refseq/). These sequences were aligned, and homology was calculated from the p-distance by ClustalW multiple alignment in MEGA 4 software. A phylogenetic tree was constructed using the MEGA 4 program, and the genetic distance was inferred using the Neighbor-Joining method [47] with 1,000 bootstrap replicates.

### Plasmid construction

Monkey podoplanin cDNA was obtained by reverse transcription polymerase chain reaction (RT-PCR) from JTC-12 cells (Riken BRC cell bank, Tsukuba, Japan). The PCR products were subcloned by Topo TA cloning (Invitrogen, Carlsbad, CA, USA) and, after sequence confirmation, cloned into pcDNA3 (Life Technologies, Carlsbad, CA, USA) or pGEX-6P-3 (GE Healthcare, Buckinghamshire, UK) plasmids using EcoRI restriction sites, resulting in pcDNA3 monkey podoplanin or pGEX-6P-3 monkey podoplanin, respectively. The pcDNA3 construct was used to generate a stable transfectant (mkyPDPN) or mutated cDNAs with PLAG deletions (mkyPDPN-ΔPLAG3, -ΔPLAG4, and -ΔPLAG3+4) using a QuikChange site-directed mutagenesis kit (Agilent Technology, Santa Clara, CA, USA) according to the manufacturer's instructions. The pGEX-6P-3 monkey podoplanin construct was used for glutathione S-transferase (GST) -tagged point mutations of cDNA using epitope mapping as described previously [48]. A human podoplanin cDNA gene was cloned into the pcDNA3 vector and a stable transfectant (hPDPN) or human podoplanin mutants with PLAG deletions (hPDPN-ΔPLAG3, and -ΔPLAG4) were generated as previously described [9, 14]. A plasmid encoding partial cDNA including the monkey podoplanin PLAG4 domain (XM_005544745.2, 409–510 b.p.; 157–170 aa; Integrated DNA Technologies, Iowa, USA) was repeatedly connected 21 times onto a pGEX-6P-3 vector and used as the immunogen to generate a monoclonal antibody (mAb) specific to the PLAG4 domain.

### Cell lines and culture conditions

CHO cells were purchased from the American Type Culture Collection (ATCC, Manassas, VA, USA) and cultured in RPMI 1640 media (Wako, Osaka, Japan) containing 10% FBS (Sigma-Aldrich, St. Louis, MO, USA). We established CHO cell lines stably transfected with vectors containing no podoplanin (CHO/mock), wild-type human podoplanin (CHO/hPDPN-WT), wild-type monkey podoplanin (CHO/mkyPDPN-WT) or the monkey podoplanin mutants with PLAG deletions (CHO/mkyPDPN-ΔPLAG3, -ΔPLAG4, and -ΔPLAG3+4) (Supplementary Figure 2A) by the same procedure as previously described [9]. The cells were cultured in medium containing 1 mg/mL of G418 (Life Technologies). JTC-12 cells (Riken BRC cell bank), derived from *Macaca fascicularis* kidneys, were cultured in DMEM low-glucose medium (Wako, Tokyo, Japan) containing 5% FBS. Vero cells (ATCC), derived from *Cercopithecus aethiops* kidneys, were cultured in DMEM medium (Wako) containing 1 mM sodium pyruvate and 10% FBS. CV-1 and COS-7 cells (ATCC), derived from *Cercopithecus aethiops* kidneys, were cultured in MEM (Wako) containing x1 MEM nonessential amino acids (Wako), 1 mM sodium pyruvate (Sigma), and 10% FBS. Hybridoma cell lines secreting anti-podoplanin antibody were cultured in RPMI 1640 media (Wako) containing 10% FBS (Sigma). DIG104.10H.1 hybridoma cells (JCRB cell bank, Osaka, Japan) were cultured in DMEM low-glucose medium (Wako) containing 10% FBS.

### Animals

Female BALB/c, BALB/c-*nu*/*nu*, and Jcl:ICR mice were purchased from Charles River (Kanagawa, Japan). All animal procedures were conducted in accordance with the guidelines of the Japanese Foundation for Cancer Research Animal Care and Use Committee. Male cynomolgus monkeys (*Macaca fascicularis*) were used for toxicity testing at the BoZo Research Center Inc. (Tokyo, Japan), conducted by the center's Institutional Animal Care and Use Committee.

### Flow cytometric analysis

To analyze podoplanin expression, cells were harvested and treated with 1 μg/mL of anti-podoplanin antibodies (Sigma-Aldrich), followed by incubation with Alexa Flour 488-conjugated anti-mouse IgG (H+L) (Thermo Fisher Scientific, Waltham, MA, USA). For CLEC-2 binding analysis, cells were incubated with 0.4 to 1 μg/mL of (His)_10_-tagged human CLEC-2 or 5 μg/mL of (His)_10_-tagged mouse CLEC-2 (R & D Systems, Minneapolis, MN, USA), followed by incubation with Alexa Flour 488-conjugated anti-penta-His antibody (Qiagen, Venlo, Netherlands). For the antibody inhibition assay, cells were incubated with 100 μg/mL of anti-podoplanin mAbs or control IgG for 30 min on ice before incubation with human or mouse CLEC-2. Fluorescence intensity was measured using a Cytomics FC500 flow cytometry system (Beckman Coulter, CA, USA) and analyzed with FlowJo software (Treestar, Inc., San Carlos, CA).

### Platelet aggregation assay

Murine whole blood from cardiac puncture in Jcl:ICR mice killed with sevoflurane was mixed with heparin or 3.2% (w/v) sodium citrate solution to produce platelet-rich plasma. The platelet aggregation rate was estimated by a previously described procedure [14].

### Western blot analysis

Cells were lysed in lysis buffer (0.1 M Tris-HCl pH 7.5, 10% glycerol, and 1% sodium dodecyl sulfate [SDS]) and boiled for 5 min. Total protein concentrations were determined with BCA Protein Assay Reagent (Pierce, Rockford, IL, USA). Each cell lysate or GST-tagged mkyPDPN-WT and its point mutants extracted from BL21 (DE3) *E. coli* (Thermo Fisher Scientific) were treated with SDS sample buffer, electrophoresed on an SDS-polyacrylamide gel (Nacalai Tesque), and transferred onto PVDF membranes (EMD Millipore, MA, USA). The membranes were incubated with primary antibodies to podoplanin (D2-40 [M3619, Dako, CA, USA], MS-1, PG4D2, 1F6, 2F7, and 3F4), β-actin (clone, AC-15, Sigma-Aldrich), or GST (clone 4C10, ab73934, Abcam, Cambridge, UK), followed by treatment with horseradish peroxidase-conjugated anti-mouse IgG (RPN2232, GE Healthcare), and finally reacted with ECL Prime Western Blotting Detection reagent (GE Healthcare). The proteins were visualized with enhanced chemiluminescence by an Amersham Imager 600 (GE Healthcare).

### Production and purification of anti-PLAG4 monoclonal antibodies

A monkey podoplanin cDNA region encoding amino acids 76 to 89 (226–267 bp) was tandemly connected 21 times in a pGEX-6P-3 vector (GE Healthcare), replicated in BL21 (DE3) *E. coli* as the GST-tagged monkey podoplanin peptide (76–89 aa), and purified using glutathione sepharose (GE Healthcare). Six-week-old female BALB/c mice were immunized by neck and ventral subcutaneous injections of the GST-tagged peptide with Titer MAX Gold adjuvant (Titer MAX, Norcross, GA, USA). Cell fusion was performed by the procedure described previously [14]. Positive clones were subcloned three times by limiting dilution, and some were purified from ascites as described previously [48]. For the acute toxicity test with cynomolgus monkeys, large-scale purification of control IgG1 and 2F7 antibody was conducted. Six-week-old female BALB/c-*nu*/*nu* mice were injected intraperitoneally with 2F7-secreting hybridomas or control IgG1-secreting hybridomas (DIG104.10H.1) suspended in Hanks’ Balanced Salt Solution (HBSS, Gibco). Ascitic fluid was collected for purification of the antibody by salting out with ammonium sulfate and performing affinity column chromatography with protein G using AKTA Explorer 10S (GE Healthcare). The IgG isotype was determined with a Mouse Monoclonal Antibody Isotyping Test Kit (AbD Serotec, Oxford, UK).

### Inhibitory effect of anti-podoplanin antibodies on pulmonary metastasis

Six-week-old female BALB/c-*nu*/*nu* mice were intravenously injected with anti-podoplanin mAbs (10 μg/mouse) through the lateral tail vein the day before cell injection. CHO/mock or CHO/mkyPDPN-WT cells were suspended in HBSS (Gibco) and injected intravenously (2.5 × 10^5^ cells/mouse) through the lateral tail vein. The mice were killed 16 days later. The lungs were removed and stained with saturated picric acid solution. The numbers of metastatic foci on the lung surface were counted.

### Quantitative reverse transcription polymerase chain reaction

Quantitative reverse transcription polymerase chain reaction (qRT-PCR) was performed using a FastStart Essential DNA Green Master (Roche, Basel, Switzerland). A Monkey Tissue cDNA Panel (Zyagen) was used to screen for monkey *pdpn* and *β-actin* expression. Standard curves were generated from a dilution series of cDNA from lymph nodes. Primer pairs used in qRT-PCR were as follows: monkey *pdpn* forward, 5’-AAATGTCAGGAAGGTACTCG-3’ and reverse, 5’-GCCGGGCAAGTGTTCCAC-3’; monkey *β-actin* forward, 5’-CCAACCGCGAGAAGATGA-3’ and reverse, 5’-CCAGAGGCGTACAGGGACAG-3’.

### Toxicity test

Male cynomolgus monkeys (*Macaca fascicularis*) (each group has N=2), which were 41 to 44 months of age and 3.64 to 4.22 kg of body weight, were intravenously injected with 100 mg/kg of 2F7 antibodies or control IgG1 through the cephalic vein. Blood was collected 7 days before and 6 and 13 days after the injection for hematology and biochemistry analysis. The monkeys were euthanized 2 weeks after injection and tissues and organs were weighed. Tissue sections were fixed in 10% neutral buffer formalin and stained with hematoxylin-eosin. Analysis of tissue specimens was performed by two independent pathologists (Drs. Kanda and Furuta) who were blind to diagnosis.

### Immunohistochemical staining of anti-podoplanin antibodies

To estimate the reactivity of 2F7 to each monkey tissues, we compared the reactivity of 2F7 with the commercially available antibody to human podoplanin. Immunohistochemical staining was performed on paraffin sections using a polymer peroxidase method. Briefly, deparaffinized and rehydrated sections were activated antigenicity of antigen in EnVision FLEX TARGET RETRIEVAL SOLUTION High pH (Dako) for 45 min at 95°C and cooled for 30 min. Sections were treated with 3% hydrogen peroxide in methanol for 5 min to block endogenous peroxidase activity. After rinsing in PBS, sections were incubated with primary antibodies to podoplanin (2F7, D2-40 [Dako], FL-162 [sc-134482, Santa Cruz Biotechnology, CA, USA], NZ-1 [012-25863, Wako], LpMab-12 [MABT841, EMD Millipore] or control IgG) overnight. Thereafter, they were incubated with the Histofine Simple Stain MAX PO (MULTI) kit (Nichirei, Tokyo, Japan) for 30min. Sections were stained with 3,3-diaminobenzidine tetrahydrochloride and counterstained with hematoxylin.

### Statistical analysis

The Mann–Whitney *U*-test was performed to determine the statistical significance of comparisons. Statistical significance was assumed for ^*^P < 0.05 or ^**^P < 0.01. All statistical tests were two-sided.

## SUPPLEMENTARY MATERIALS FIGURES


