# Supplementary material for: A safety study of newly generated anti-podoplanin-neutralizing antibody in cynomolgus monkey (Macaca fascicularis)
Source: Oncotarget. 2018 Sep 7;9(70):33322–36. doi: 10.18632/oncotarget.26055 (PMC6161800; doi:10.18632/oncotarget.26055)
Supplement: Supplementary file 1 [file oncotarget-09-33322-s001.pdf]

A safety study of newly generated anti-podoplanin-neutralizing antibody in cynomolgus monkey (*Macaca fascicularis*)

SUPPLEMENTARY MATERIALS

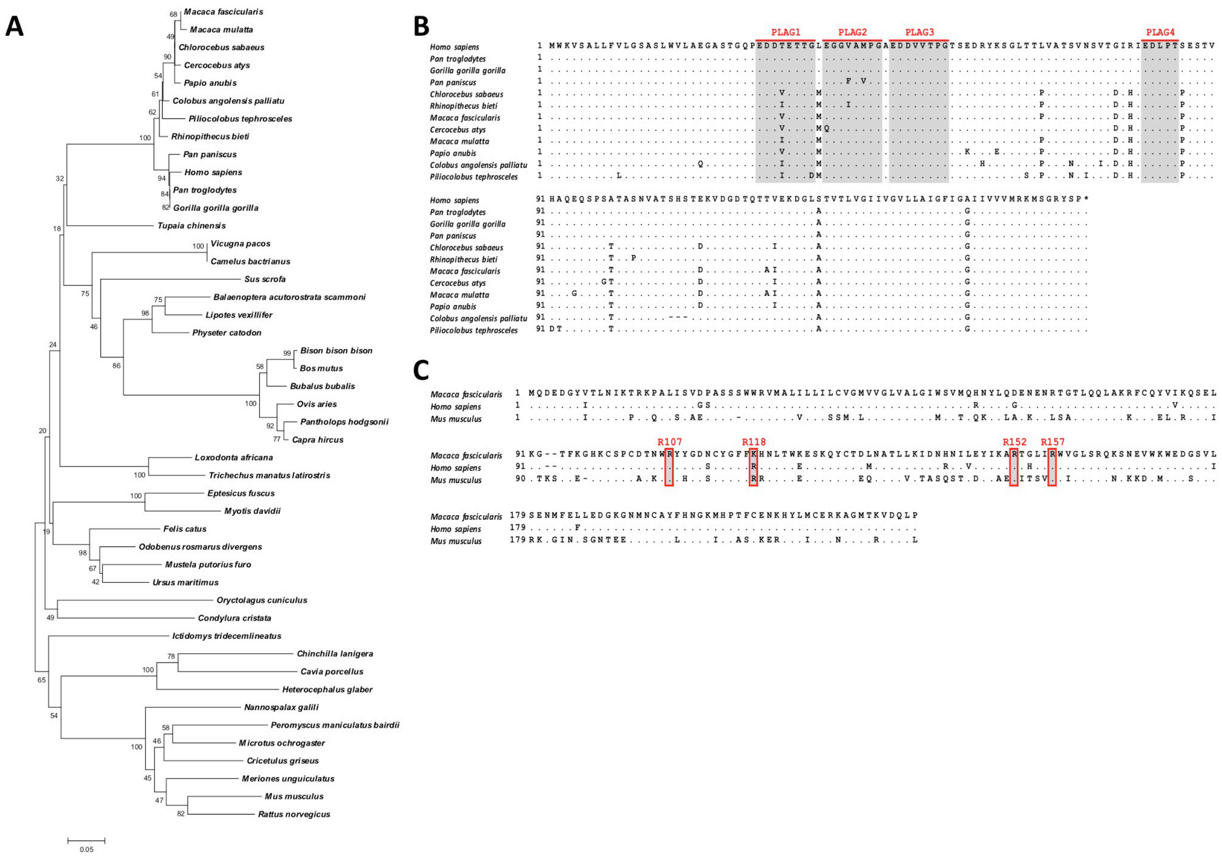

Supplementary Figure 1: Phylogenetic relationships of podoplanin or CLEC-2 protein sequences. **(A)** Phylogenetic relationships of podoplanin (PDPN). The tree was constructed using amino acid sequences aligned by ClustalW. GenBank accession numbers: *Homo sapiens*, NM_006474.4; *Pan troglodytes*, XM_001148148.6; *Gorilla gorilla gorilla*, XM_004024702.2; *Pan paniscus*, XM_003806267.2; *Chlorocebus sabaeu*, XM_007980479.1; *Rhinopithecus bieti*, XM_017870446.1; *Macaca fascicularis*, XM_005544745.2; *Cercocebus atys*, XM_012077051.1; *Macaca mulatta*, XM_015114458.1; *Papio anubis*, XM_003891148.4; *Colobus angolensis palliatus*, XM_011939929.1; *Piliocolobus tephrosceles*, XM_023195279.1; *Tupaia chinensis*, XM_006145278.2; *Sus scrofa*, XM_005665017.3; *Camelus bactrianus*, XM_010957510.1; *Vicugna pacos*, XM_015238964.1; *Balaenoptera acutorostrata scammoni*, XM_007174935.1; *Lipotes vexillifer*, XM_007463182.1; *Physeter catodon*, XM_007104824.2; *Bison bison bison*, XM_010850060.1; *Bos mutus*, XM_005889789.2; *Bubalus bubalis*, XM_006074459.1; *Ovis aries*, XM_004013802.3; *Capra hircus*, XM_005690821.3; *Pantholops hodgsonii*, XM_005966287.1; *Eptesicus fuscus*, XM_008148201.1; *Myotis davidii*, XM_015565873.1; *Condylura cristata*, XM_004679333.2; *Trichechus manatus latirostris*, XM_012555799.2; *Loxodonta africana*, XM_010593104.2; *Felis catus*, XM_006934300.4; *Ursus maritimus*, XM_008694703.1; *Odobenus rosmarus divergens*, XM_012561235.1; *Mustela putorius furo*, XM_004741440.2; *Oryctolagus cuniculus*, XM_008275367.2; *Ictidomys tridecemlineatus*, XM_005317481.3; *Chinchilla lanigera*, XM_005409891.2; *Cavia porcellus*, XM_013154175.2; *Heterocephalus glaber*, XM_004850314.3; *Nannospalax galili*, XM_008856054.2; *Rattus norvegicus*, NM_019358.1; *Mus musculus*, NM_010329.3; *Mesocricetus auratus*, XM_021233536.1; *Cricetulus griseus*, XM_007607929.1; *Meriones unguiculatus*, XM_021650251.1; *Peromyscus maniculatus bairdii*, XM_006975446.2; *Microtus ochrogaster*, XM_005352878.2. **(B)** PDPN protein sequences of humans and of various monkey species. **(C)** Monkey, human, and mouse C-type lectin-like receptor 2 protein sequences. GenBank accession numbers: *Macaca fascicularis*, XM_005570100.1; *Homo sapiens*, NM_016509.3; *Mus musculus*, NM_019985.3.

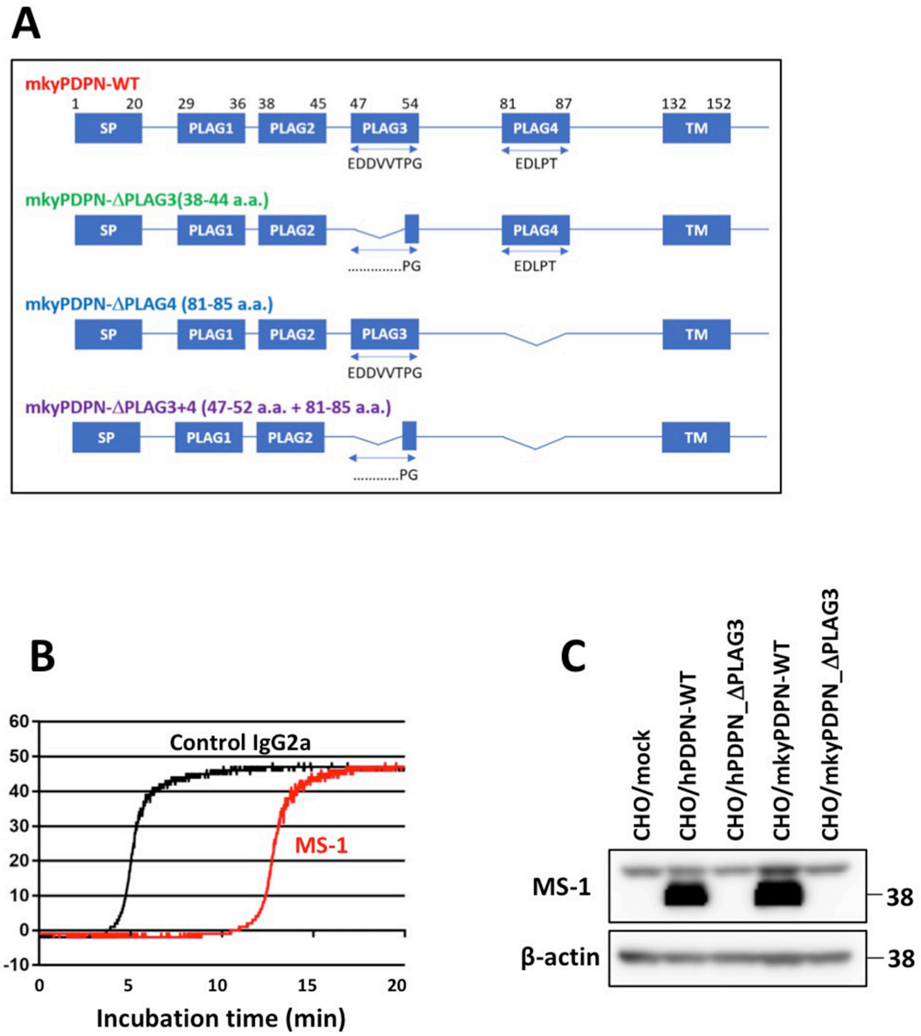

Supplementary Figure 2: Schematic representation of PLAG domain-deleted mutants and cross-reactivity of MS-1 antibody to monkey podoplanin. (A) Schematic representation of wild-type monkey podoplanin (mkyPDPN-WT) and PLAG domain-deleted mutants (mkyPDPN−ΔPLAG3, −ΔPLAG4, and −ΔPLAG3+4). **(B)** CHO cells transfected with mkyPDPN-WT were incubated with 10 µg/mL of control IgG2a or the PLAG3-specific antibody MS-1, followed by incubation with mouse platelet-rich plasma to measure platelet aggregation. **(C)** CHO transfected with empty vector (mock), hPDPN-WT, hPDPN−ΔPLAG3, mkyPDPN, or mkyPDPN−ΔPLAG3 cells were lysed and immunoblotted with MS-1 antibodies to PDPN or with β-actin.

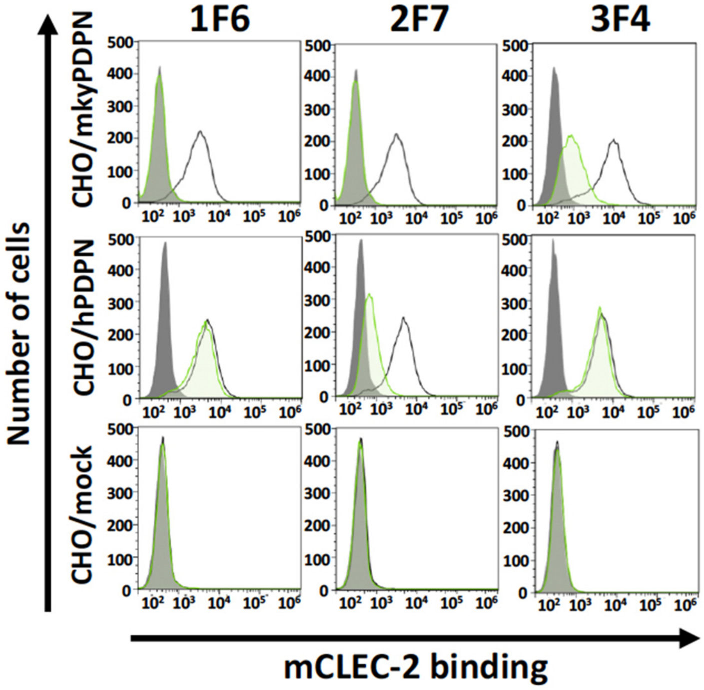

Supplementary Figure 3: The suppressive effect of anti-podoplanin antibodies to mouse CLEC-2. CHO cells transfected with an empty vector (mock), wild-type monkey podoplanin (mkyPDPN-WT), or wild-type human podoplanin (hPDPN-WT) were incubated with 100 μg/mL of control IgG1 or anti-podoplanin antibodies 1F6, 2F7, or 3F4, followed by incubation with 5 μg/mL of mouse C-type lectin-like receptor 2 [mCLEC-2-(His)$_{10}$] (open areas: control IgG-treated samples; green areas: anti-PDPN mAb-treated samples). After washing, cells were further incubated with Alexa Flour 488-conjugated anti-penta-His second antibody. CLEC-2 binding was measured by flow cytometry. Gray areas indicate the fluorescence intensity of samples not treated with CLEC-2.

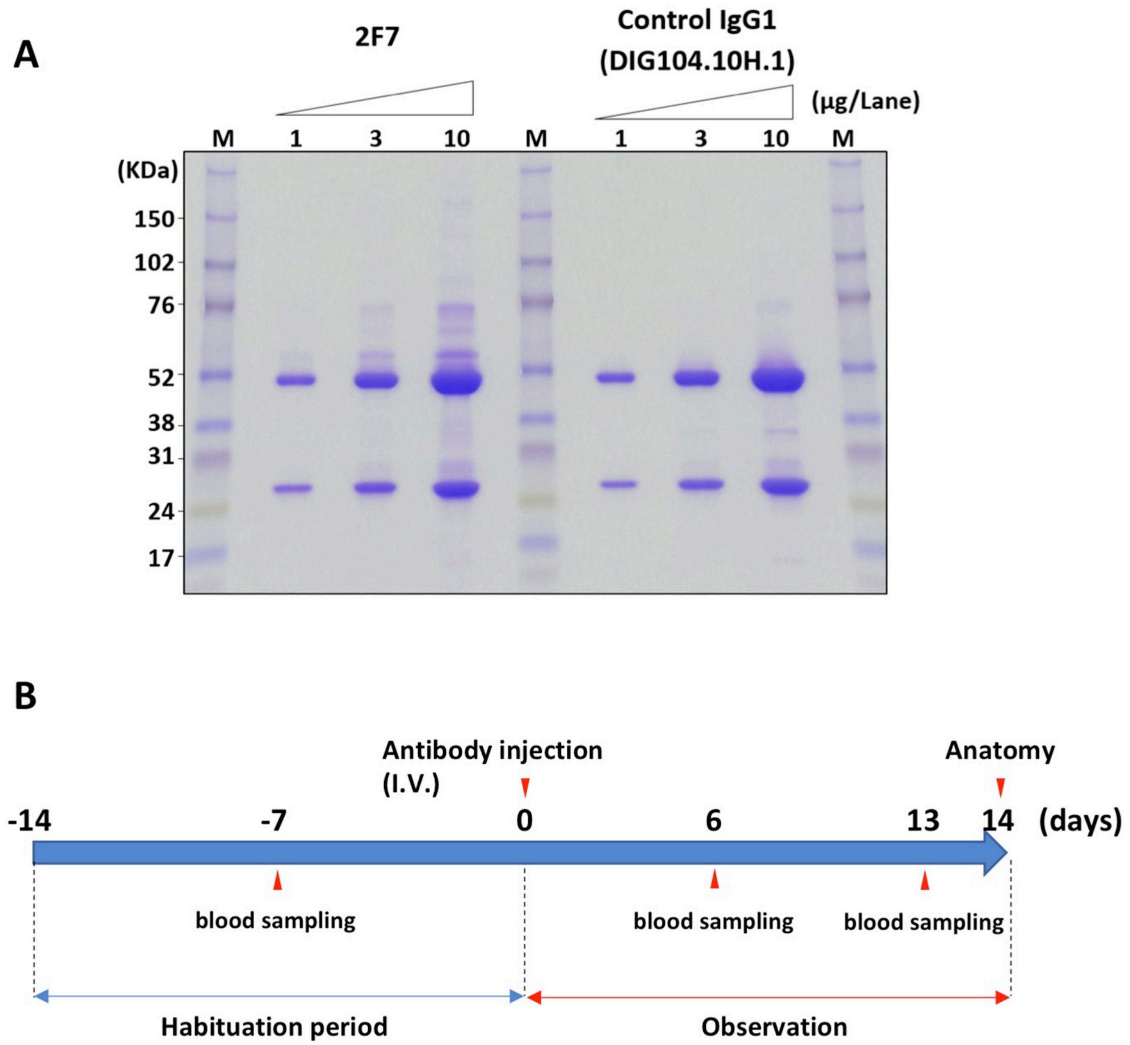

Supplementary Figure 4: Large-scale purification of 2F7 antibody and control IgG1 for acute toxicity test and outline of acute toxicity test. **(A)** Large-scale production of the anti-podoplanin neutralizing antibody 2F7 was performed by collecting ascitic fluid from BALB/c-*nu/nu* mice secreting 2F7 or DIG104.10H.1 as the control IgG1. Antibody purity was estimated with Coomassie Brilliant Blue stain. **(B)** Outline of acute toxicity testing with cynomolgus monkeys.

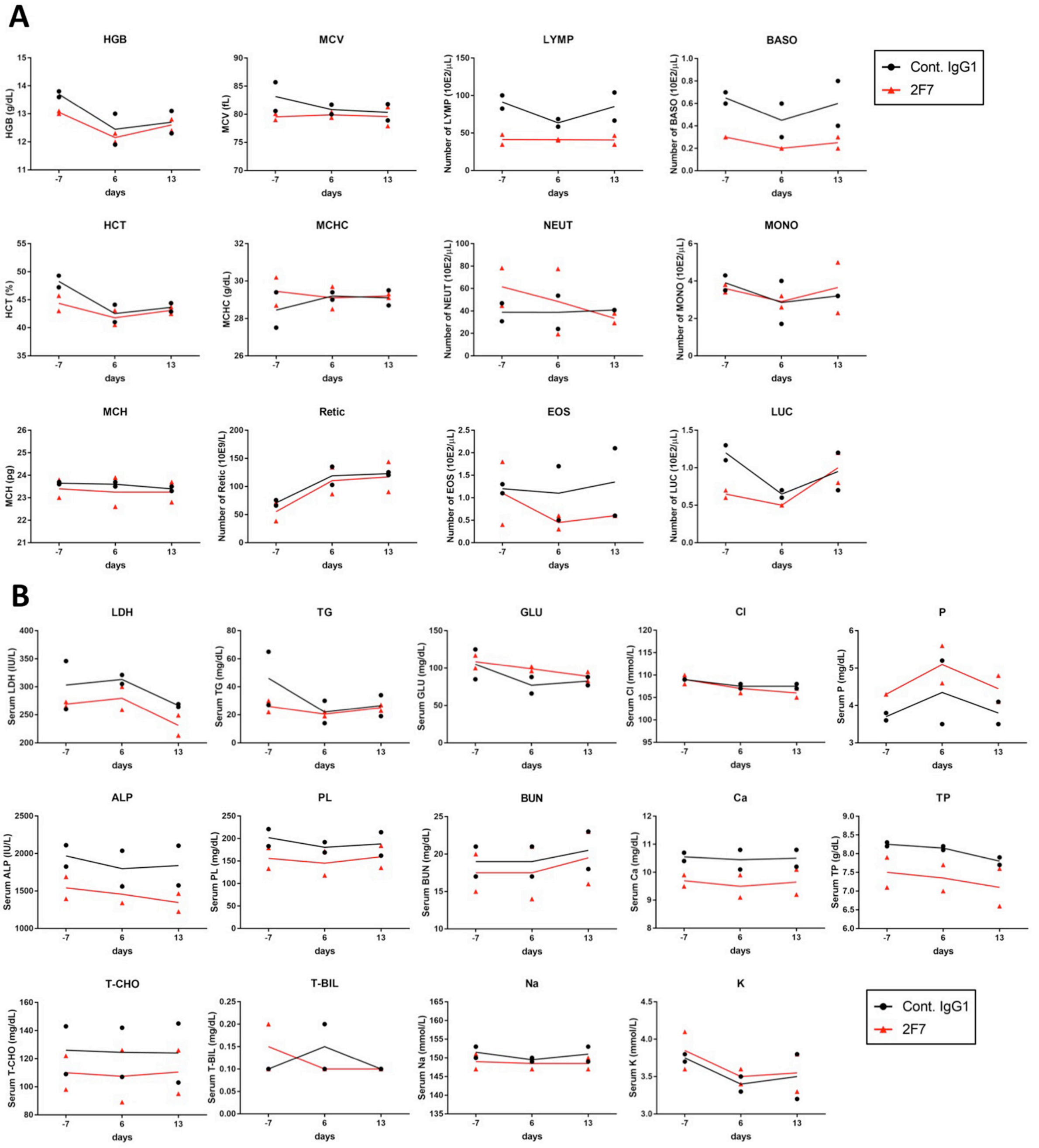

Supplementary Figure 5: Hematologic and biochemical analysis of blood samples. Monkeys were intravenously injected with 100 mg/kg of control IgG1 or 2F7 antibodies for toxicity testing. Blood was collected 7 days before and 6 and 13 days after injection for **(A)** hematology and **(B)** biochemistry analyses. Normal range for each parameter was shown as mean (-2SD/ +2SD). (A) HGB (hemoglobin), 13.5 (11.8/ 15.1) g/dL; HCT (hematocrit), 44.5 (38.3/ 50.8) %; MCH (mean corpuscular hemoglobin), 23.4 (21.2/ 25.6) pg; MCV (mean corpuscular volume), 77.4 (70.1/ 84.8) fL; MCHC (mean corpuscular hemoglobin concentration), 30.3 (28.0/ 32.5) g/dL; Retic (reticulocyte), 81.8 (29.0/ 134.6) 10E9/L; LYMP (lymphocyte), 60.5 (9.4/ 111.5) 10E2/μL; NEUT (neutrophil), 51.2 (0.0/ 108.6) 10E2/μL; EOS (eosinophil), 0.8 (0.0/ 4.5) 10E2/μL; BASO (basophil), 0.5 (0.0/ 1.6) 10E2/μL; MONO (monocyte), 3.3 (0.1/ 6.5) 10E2/μL; LUC (leucocyte), 0.6 (0.0/ 1.8) 10E2/μL. (B) LDH (lactase dehydrogenase), 339 (157/ 522) IU/L; ALP (alkaline phosphatase), 1687 (673/ 2701) IU/L; T-CHO (total-cholesterol), 132 (74/ 190) mg/dL; TG (triglyceride), 24 (0/ 51) mg/dL; PL (phospholipid), 177 (109/ 246) mg/dL; T-BIL (total-bilirubin), 0.2 (0.0/ 0.4) mg/dL; GLU (glucose), 85 (46/ 124) mg/dL; BUN (blood urea nitrogen), 20 (11/ 29) mg/dL; Na (sodium), 152 (145/ 159) mmol/L; Cl (chlorine), 109 (103/ 114) mmol/L; Ca (calcium), 10.0 (9.2/ 10.8) mg/dL; K (potassium), 4.0 (3.0/ 4.9) mmol/L; P (phosphorus), 5.0 (3.1/ 7.0) mg/dL; TP (total-protein), 7.5 (6.6/ 8.4) g/dL.

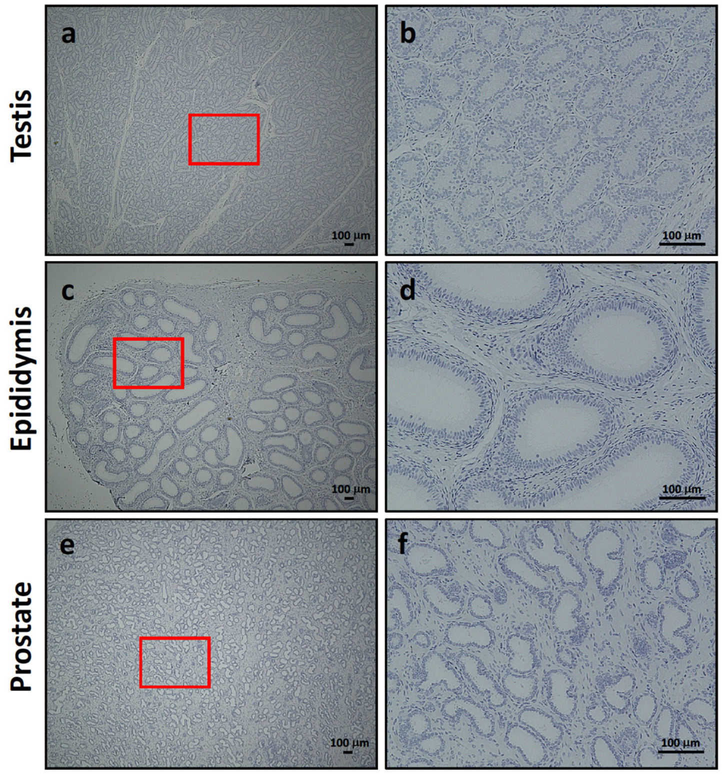

Supplementary Figure 6: Immunohistochemical staining of monkey tissue samples using control IgG1. Representative image of podoplanin expression in testis, epididymis, prostate was shown. Each section of monkey tissues was incubated with control IgG1 and counterstained with hematoxylin. Testis **(a** and **b)**, epididymis **(c** and **d)**, or prostate **(e** and **f)**. The higher magnification of area surrounded by red line (a, c or e) was shown in b, d, or f, respectively.
